# Sustaining Improvements in CLABSI Reduction in a Pediatric Cardiac Intensive Care Unit

**DOI:** 10.1097/pq9.0000000000000575

**Published:** 2022-06-23

**Authors:** Jennifer Gauntt, Sarah Brandt, Kevin Dolan, Jaime Manley, Roxann Tyner, Wendi Beauseau, Janet M. Simsic

**Affiliations:** From the * The Heart Center at Nationwide Children’s Hospital, Columbus, Ohio; † Center for Clinical Excellence at Nationwide Children’s Hospital, Columbus, Ohio; ‡ Department of Quality Improvement Services at Nationwide Children’s Hospital, Columbus, Ohio.

## Abstract

Central line-associated bloodstream infections (CLABSIs) are preventable events that increase morbidity and mortality. The objective of this quality project was to reduce the incidence of CLABSIs in a pediatric cardiothoracic intensive care unit.

**Methods:**

Institutional review of an unacceptably high rate of CLABSIs led to the implementation of 4 new interventions. These interventions included: the use of sequential cleaning between line accesses, Kamishibai card audits, central line utilization and entry audits, and proctored simulation of line access.

**Results:**

There was a reduction in CLABSI rate from 1.52 per 1,000 central line days in 2018 to 0.37 per 1,000 central line days in 2020 and 0.32 in 2021. Additionally, central line days per 100 patient days decreased from 77 to 70 days over the study period. The cardiothoracic intensive care unit went 389 days without a CLABSI from October 2020 to November 2021.

**Conclusions:**

Implementation of multiple interventions led to a successful reduction in the incidence of CLABSIs in our unit, with a sustained reduction over 1 year.

## INTRODUCTION

Central line-associated bloodstream infections (CLABSIs) are a major source of hospital-acquired infections and are associated with increased morbidity, mortality, and costs.^1,2^ Studies have demonstrated that implementing bundle strategies, including insertion and maintenance bundles, reduces CLABSI rates.^3–5^ In pediatrics, Children’s Hospitals’ Solutions for Patient Safety (SPS) CLABSI bundle, guidelines followed by over 135 children’s hospitals, has also been shown to reduce CLABSI rates across network hospitals.^6^ Adequate disinfection of the catheter hub before line entry to prevent intraluminal contamination is a key component of the central line maintenance bundle.^7^ Suitable disinfection can be achieved by scrubbing the hub for a predetermined time (10–20 s) followed by a specified drying time before and after each line entry.^8^ Several single-center studies have demonstrated that a “scrub the hub” procedure decreased the incidence of catheter-related sepsis in neonates.^9,10^

Novel strategies from the business world have also been used in healthcare to improve safety. Kamishibai cards (K-Cards), used by Toyota as an auditing management tool, provide standardization for real-time, peer-to-peer feedback between workers and auditors.^11^ K-Cards are useful to minimize differences between auditors, resulting in reduced variability and enhanced attention to detail.^11^ Multiple studies have demonstrated an association between K-Card utilization and increased healthcare bundle compliance. The interaction between auditor and clinician serves as a reminder of the bundle elements and is an opportunity for real-time feedback.^12,13^

Simulation is an educational strategy adapted to the healthcare world with success.^14–16^ Healthcare simulation provides learners with realistic clinical situations and allows them to practice and perfect in a safe environment. An instructor provides real-time guidance and feedback to participants during the simulation. Clinical personnel are recommended to undergo continual systematic training, rehearsal, performance assessment, and refinement in their practice to maintain competency and improve the quality and safety of care.^17^

This quality project aimed to reduce the rate of CLABSIs in a pediatric cardiothoracic intensive care unit (CTICU) by implementing 4 new interventions to the existing CLABSI bundle.

## METHODS

### Ethical Issues

This quasi-experimental quality improvement endeavor involved the development of new processes to reduce CLABSIs. No interventions involved comparing multiple devices or therapies, and patients were not randomized. Healthcare staff and quality improvement team members accessed medical records as part of their normal responsibilities. The institutional review board did not deem this project human subjects research, precluding review per hospital policy.

### Setting

Nationwide Children’s Hospital is an academic, nonprofit, freestanding children’s hospital located in Columbus, Ohio. The CTICU is a 20-bed unit with over 600 admissions per year. The CTICU staff includes a multidisciplinary team of critical care and cardiology physicians (n = 10), advanced nurse practitioners (n = 17), a dedicated clinical pharmacist (n = 1), registered nurses (n = 75), respiratory therapists (n = 14), and pediatric cardiology and pediatric critical care physicians in fellowship training (n = 21). In addition, dedicated clinical dietitians, physical and occupational therapists, child life specialists, and social workers round out the clinical team.

### Planning the Interventions

**Fig. 1.**
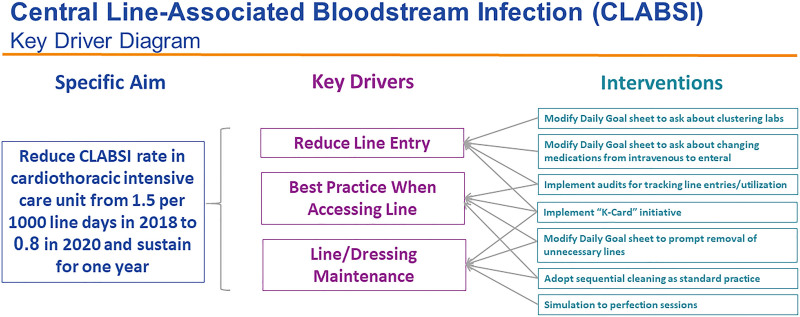
Key driver diagram.

We recruited a multidisciplinary team to develop and implement standardized interventions for CLABSI reduction in the CTICU. The team included CTICU physicians, a nurse leader from the Department of Epidemiology, CTICU advanced nurse practitioners, CTICU nurses, and our quality improvement service line coordinator. The first steps were to create an aim statement (to reduce the CLABSI rate in the CTICU from 1.52 per 1,000 line days in 2018 to 0.8 per 1,000 line days in 2020 and sustain it for 1 y) and a key driver diagram (Fig. 1). We identified reduced line entry, best practice when accessing lines, and line/dressing maintenance as the relevant key drivers.

### Interventions

Hospital-wide interventions have been introduced over the years in a continued attempt to reduce CLABSIs. The interventions introduced seemed to have some short-term benefits. However, CLABSIs continued to occur in the CTICU at unacceptable rates over the years despite near-perfect compliance with the SPS CLABSI bundle (97%–99% between 2018 and 2021), which our Epidemiology Department tracked monthly. Therefore, the CTICU leadership sought to explore additional interventions to achieve a sustained reduction in CLABSIs. As a result, a multidisciplinary team from our CTICU visited a high-functioning program with a similar census and case mix and a low CLABSI rate to explore potential shortcomings in our program. Due to this interfacility collaboration and additional intrafacility multidisciplinary meetings, we instituted the following interventions.

### Sequential Cleaning

Previous CLABSI huddles identified frequent line entries as a risk factor for catheter hub contamination and increased CLABSI rates. Therefore, we implemented sequential cleaning at the bedside in April 2019 and added sequential cleaning to nursing biannual simulation competencies in September 2019. Sequential cleaning involves scrubbing the needleless connector/hub with an alcohol scrub device for the defined period (Site Scrub—10 s of a twisting motion, alcohol prep pad—15-s scrub with a 5-s dry) before any connection. The nurse repeats the scrub before each line entry (each connection of a flush, medication, or tubing). For example,

Step 1. Scrub the hub, then flush the line.

Step 2. Scrub the hub, then give the medication.

Step 3. Scrub the hub, then flush the line.

Step 4. Scrub the hub, then give the second medication.

Step 5. Scrub the hub, flush the line, then apply alcohol impregnated cap at the end of the sequential cleaning process.

In our institution, nurses primarily use an alcohol prep pad to clean the outside of the tubing before any disconnection, and they clean the tubing spike when changing an intravenous (IV) bag. In addition, nurses primarily use Site Scrub before any needleless connector entry.

### K-Card Audits

K-Cards (**see figure S1**, **Supplemental Digital Content 1**, which describes CLABSI K-card audit form, http://links.lww.com/PQ9/A384) were implemented in April 2019 to audit CLABSI bundle compliance. Initially, the goal was for members of the nursing clinical leader team and unit CLABSI champions to complete 20 K-Cards per month (goal of 180 audits in 2019 and 240 audits in 2020). However, starting in June 2021, the goal K-Card audit frequency increased to 1 audit every 12-hour nursing shift (ie, 2 per day or 480 total for 2021). A unit-based nurse in a clinical leadership role randomly selects a patient’s bedside nurse with a central line and performs the CLABSI K-Card audit. These 2 nurses discuss the following elements related to CLABSI reduction:

(1) Daily Goals—did the team discuss the necessity of the central line, consolidation of line entries, site integrity, and mechanical issues during rounds today?

(2) Central Line Dressing—visually confirm that the central line dressing is clean, dry, occlusive, and secured appropriately. In addition, visually confirm that the dressing change date is appropriately documented in the medical record and not past due.

(3) IV Tubing—visually confirm that all IV tubing is dated appropriately and not past due. Also, visually confirm that the alcohol port protectors (cap) are present on all injection sites.

(4) Needleless Connector—visually confirm that the needleless connector is present and that the change date is appropriately documented in the medical record and not past due.

(5) Line Access Observation—observe the bedside nurse completing the following steps when entering the central line or observe the bedside nurse simulating the procedure: hand hygiene and clean gloves worn immediately before line-entry; use of sterile drape; alcohol port protector removed and hub scrub appropriately completed before line-entry; hub scrubs appropriately completed before each entry into the central line (sequential cleaning); aseptic technique maintained throughout the procedure.

(6) Bath—ask the bedside nurse if the patient received an age-appropriate bath and confirm it was documented in the medical record.

The auditor was given nonpunitive scripting for reminders and prompting of line entry elements. However, the auditor did not record the identity of the audited nurse; therefore, there was no mechanism to prevent the same nurse from being audited multiple times.

### Central Line Utilization and Entry Audits

Bedside nurses tracked line entries for every patient with a central line for 24 hours every month from June 2019 to October 2020. Each nurse completed a tracking sheet (**see figure S2**, **Supplemental Digital Content 1**, which describes CLUE data collection form. CVL, central venous line; ECMO, extracorporeal membrane oxygenation; PICC, peripherally inserted central catheter; RA, right atrial; UVC, umbilical venous catheter, http://links.lww.com/PQ9/A384) to document all line entries for their 12-hour shift and recorded the purpose of the line entry as for laboratory collection or another purpose (eg, medication administration). The results of this audit were shared with the medical team the following day on morning rounds to increase awareness regarding the number of line entries per patient per day and guide the discussion to determine:

(1) Are the central lines still needed?

(2) Can labs be consolidated or obtained less frequently?

(3) Can medications be switched from IV to enteral?

The goal of these central line utilization and entry (CLUE) audits and subsequent discussions during rounds was to prompt the medical team to reduce line entries and remove unnecessary lines if deemed clinically appropriate.

### Line Maintenance and Line Access Simulations

CTICU nurses undergo simulation training of specific nursing tasks twice per year, called “simulation to perfection” in our institution. In September 2019, nursing leadership added line maintenance and line access to the “simulation to perfection” training to assure competency surrounding the processes of central line dressing changes, sterile line setup, and line entries. During these sessions, all staff nurses participate in simulation utilizing flat “skins” or simulation dolls with central lines. Using these tools, all nursing staff perform a sterile line setup and a timed sequential cleaning line entry to ensure the correct technique and appropriate timing of each needleless connector scrub. The unit nurse educator or clinical leader observes the simulations and provides feedback during and after the simulations (**see figure S3**, **Supplemental Digital Content 1**, which describes simulation observer assessment form, http://links.lww.com/PQ9/A384). All staff nurses (ie, nurses with an assignment to the CTICU) were required to participate twice yearly and were mandated to repeat the simulation until achieving competency. Nonstaff nurses (those who float to our unit from another intensive care unit) demonstrate line maintenance and access competency via “simulation to perfection” annually. Travel nurses assigned to the CTICU for their 6-week assignment demonstrate competency at the time of hiring. On average, no more than 10%–15% of bedside nurses in the CTICU daily are float nurses.

### Methods of Evaluation and Analysis

We used Statistical Process Control U-charts with standard upper and lower control limits (±3 SDs) to follow monthly CLABSI rates (plotted as a function of 1,000 catheter days) and monthly central line days (plotted as a function of 100 patient days). The year 2018 was used as the process baseline. We applied Nelson’s rules^18^ for special cause variation to identify a shift in a process stage mean. K-Cards were evaluated for CLABSI bundle compliance. We considered a K-Card compliant if all 6 components of the K-Card were marked “yes” and noncompliant if 1 or more bundle elements were marked “no.”

## RESULTS

**Fig. 2.**
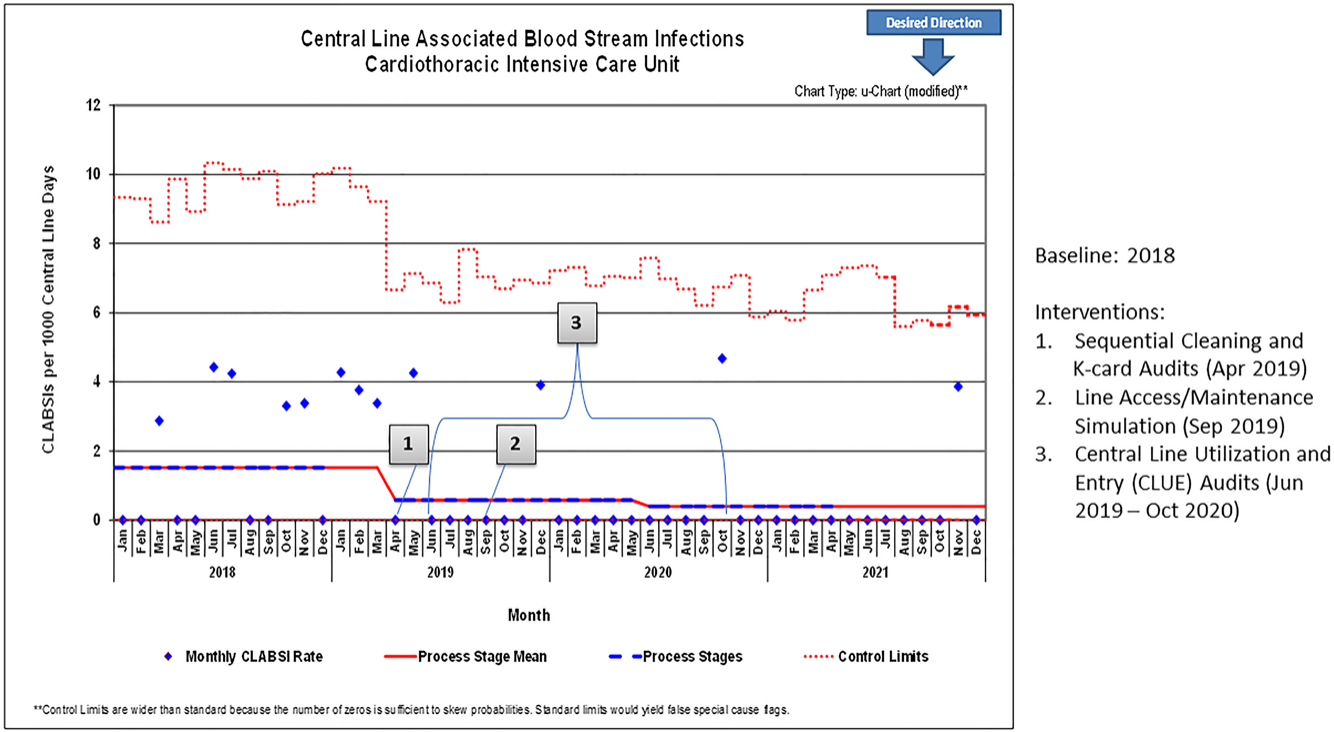
Annotated U-chart of CLABSIs per 1,000 central line days, 2018–2021.

**Fig. 3.**
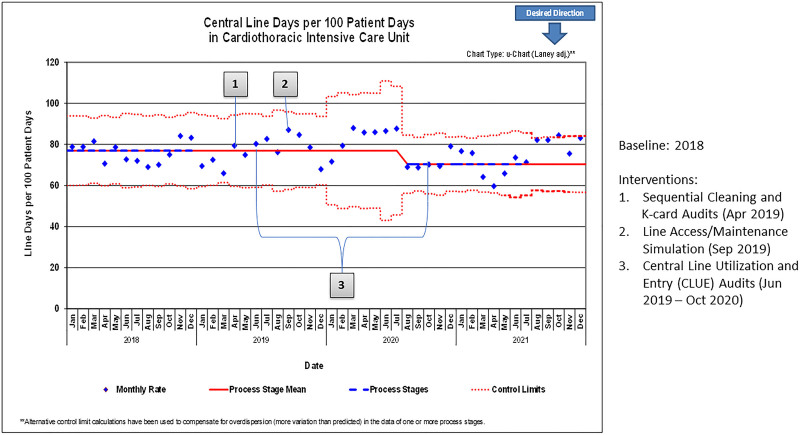
Annotated U-chart of central line days per 100 patient days, 2018–2021.

The implementation of the 4 interventions led to a decrease in the CLABSI rate from 1.52 per 1,000 central line days in 2018 to 0.37 per 1,000 central line days in 2020, sustained through 2021 (Fig. 2). There was also a reduction in adjusted central line days, from 77 to 70 per 100 patient days (Fig. 3). There was over 1 year between CLABSIs from October 2020 to November 2021.

Since the K-Card implementation in 2019, CTICU nurse leaders have completed 739 K-Cards (Table 1). In 2019, 79% of K-Cards were compliant (all 6 bundle elements were performed correctly). This result improved to 89% compliance in 2020 and 2021. The most commonly noncompliant elements identified during the K-Card audits were related to the central line dressing portion and line access observation portion of the CLABSI bundle K-Card. Nearly half of noncompliant K-Cards in 2019 were due to improper line access during observation. This improper access became less predominant after the initiation of line maintenance and access simulations via “simulation to perfection.” In addition, auditors commonly noted inadequate hub scrub time through the progression of sequential cleaning in 2019 (Table 1), although this deficiency also became less frequent after the initiation of line access simulation. There were no K-cards with more than 1 noncompliant element.

**Table 1.** K-Card Compliance

Description	2019	2020	2021
Total K-Card audits	172	236	331
Noncompliant K-Cards	36	27	35
K-Card compliance %	79	89	89
Noncompliance of individual elements, n (%)	36 (100)	27 (100)	35 (100)
Element 1—Daily goals, n (%)	2 (5.5)	1 (3.7)	1 (2.8)
Element 2—Dressing, n (%)	5 (13.8)	15 (55.6)	9 (25.7)
Element 3—IV tubing, n (%)	10 (27.8)	3 (11.1)	8 (22.8)
Element 4—Needless connector, n (%)	0	2 (7.4)	2 (5.7)
Element 5—Line access observation, n (%)	17 (47.2)	3 (11.1)	9 (25.7)
Hub scrub deficiencies included in element 5, n (%)	11 (64.7)	1 (33.3)	3 (33.3)
Element 6—Bath, n (%)	2 (5.5)	3 (11.1)	6 (17.1)

One-hundred eighty-nine CLUE audits were performed over 17 months. There was great variation in the number of line entries for patients with a central line, likely related to variation in patient acuity. For example, the number of line entries for laboratory collection in a 12-hour shift ranged from 0 to 12 entries, and the number of line entries for other reasons ranged from 0 to 16 entries.

One-hundred percent of CTICU staff nurses completed line maintenance and access simulation at each biannual session during the study period. The most commonly deficient component in the simulation was inadequate hub scrub time. The scrub time commonly decreased during the sequence of the sequential cleaning process. The donning of clean gloves before line entry was also inconsistent.

## DISCUSSION

CLABSIs are a major source of hospital-acquired infections and are associated with increased morbidity, mortality, and costs.^1,2^ Successful CLABSI reduction with sustained results requires more than bundle implementation; it requires healthcare professionals’ behavior change. Education, performance assessments, feedback, and teamwork can help accomplish this necessary behavior change.^19^ This quality project aimed to reduce and sustain the rate of CLABSI in the CTICU by implementing 4 interventions. As our compliance with SPS bundle elements was near perfect before and throughout the interventions, we believe our interventions successfully reduced and sustained the reduction in the CLABSI rate.

The interventions addressed both tangible line care deficits and healthcare provider behavior. For example, we implemented sequential cleaning and CLUE to address frequent line entries. CLUE raised awareness of the number of central line entries per patient per day. In addition to the scripted questions read by the bedside nurse during rounds, this information prompted the physician to consider central line utilization, decrease line entries, and question the need for a central line every day. Following the CLUE intervention, physicians were more likely to remove the central line, as evidenced by our decrease in central line days.

Adequate disinfection of the hub before line entry is important to prevent intraluminal contamination and is a key portion of the maintenance component of central line bundle protocols.^8^ Several single-center studies have demonstrated that a “scrub the hub” procedure decreased the incidence of catheter-related sepsis in neonates.^9,10^ Therefore, we instituted sequential cleaning, scrubbing the needleless connector/hub before each connection or individual line entry. This process can be difficult to sustain, as our audits and simulations demonstrated that the scrub time occasionally decreased during the sequence of the sequential cleaning process. However, sequential cleaning (scrubbing the hub multiple times throughout line access) leads to an overall increase in scrubbing the hub compared with traditional cleaning (scrubbing the hub once before a series of line accesses), which likely offers protection despite poor technique. Frequent audits and observations are helpful to reinforce this important process in CLABSI reduction.

We introduced K-Cards to reinforce the new process measures (as well as the existing CLABSI bundle elements) and provide real-time nonthreatening feedback. K-Cards are useful to minimize differences between auditors, resulting in reduced variability and enhanced attention to detail.^11^ There is an association between K-Card utilization and increased healthcare bundle compliance. The interaction between auditor and clinician serves as a reminder of the bundle elements and is an opportunity for real-time feedback.^12,13^ The observer addresses any irregularities in real-time, leading to enhanced awareness and hopeful reduction in missteps in the future. There is some overlap between SPS CLABSI bundle elements and K-Card elements; however, the SPS bundle generally focuses on the documentation of appropriate line maintenance, whereas the K-card bundle focuses on direct observation of line maintenance and access. This distinction is the likely cause of the difference between SPS bundle compliance and K-Card compliance in our study.

Literature supports the benefits of simulation in healthcare.^14–16^ Simulation-based learning improves skills, allows for deliberate practice for skill improvement and assessment, and leads to skill retention.^14^ Feedback during simulation is important for effective learning and should be guided by the individual’s learning needs.^14,15^ Not only does feedback improve the learner’s performance, but it also can identify the cause of variance between observed and expected actions.^14^ Feedback that includes positive reinforcement of correct performance is as important as noting the undesired actions.^14^ The learner can then reflect on both the positive and negative aspects of their performance.^20^ We implemented line maintenance and line access simulation to increase awareness and competency surrounding the processes of sterile line setup and line entries. The simulation allowed the learner to focus on the specific task in a controlled setting while receiving coaching and feedback from an expert observer. Evidence shows that simulation-based healthcare education with deliberate practice leads to improved and lasting results.^16^

K-card audits and “simulation to perfection” were tools implemented to change the behavior of healthcare professionals through performance assessments, real-time feedback, teamwork, and reinforcement of a safety culture.

## LIMITATIONS

We utilized a quality improvement methodology to complete this project, and therefore there was no control group. Multiple interventions were undertaken simultaneously with no attempt to ascertain which intervention was the most or least effective and no ability to control for the Hawthorne effect (alteration of nurse behavior during an audit simply because she was being observed). All staff nurses (100%) were required to complete the line maintenance and line access simulations. However, there was no record of the number of times a nurse may have needed to repeat the simulation to attain competency. Although central line days decreased because of CLUE audits, it was difficult to ascertain whether line entries were reduced, given our inability to control patient acuity.

## CONCLUSIONS

We believe that our 4 interventions were key components to reducing CLABSIs in our CTICU. Line maintenance and line access simulations, K-Card audits, and CLUE audits served to bring appropriate use and care of central lines to the forefront of the minds of the CTICU staff, making every team member a CLABSI-reduction champion.

## DISCLOSURE

The authors have no financial interest to declare in relation to the content of this article.

## Supplementary Material


