# Supplementary material for: Sustaining Improvements in CLABSI Reduction in a Pediatric Cardiac Intensive Care Unit
Source: Pediatr Qual Saf. 2022 Jun 23;7(4):e575. doi: 10.1097/pq9.0000000000000575 (PMC9225588; doi:10.1097/pq9.0000000000000575)
Supplement: Supplementary file 1 [file pqs-7-e575-s001.pdf]

SDC, Figure 1

CLABSI Bundle K-card		
**Observation:** Identify an RN caring for a patient with a central line and together answer all questions.		
**1. Daily Goals: Yes or No**		
	•	Ask if line necessity, consolidation of line entries, site integrity, & mechanical issues were discussed on rounds today. If rounds have not occurred, was it discussed the previous day or passed in report?
**2. Central line dressing: Yes or No**		
	•	Ask the RN if the central line dressing is clean/dry/occlusive and secured appropriately. Visually confirm the dressing is clean/dry/occlusive and secured appropriately.
	•	Ask the RN to show you where the dressing change date is documented in the medical record and not past due.
	•	Ask them the purpose of the Dressing Change Bundle Checklist.
	•	Ask them when they should complete the checklist.
**3. IV Tubing: YES or NO**		
	•	Ask the RN to show you that all IV tubing is dated appropriately & not past due. Answer "Yes" if the line is not in use.
	•	Are alcohol port protectors present on all luer injection sites?
**4. Needleless connector: YES or NO**		
	•	Ask the RN to show you where the needleless connector change date is documented in the medical record and not past due.
**5. Line access observation: YES or NO**		
	**Observe RN complete the steps below when entering the line or have RN simulate the procedure**	
	•	Hand Hygiene performed & clean gloves worn immediately prior to entering line
	•	Sterile drape used.
	•	Alcohol port protector removed & Hub scrub done prior to entering line.
	•	Hub scrubs prior to EVERY entry into the central line.
	•	Maintains aseptic technique throughout procedure
**6. Bath: YES or NO**		
	•	Ask the RN if the patient received an age-appropriate bath. Confirm that it was documented. (CHG Exclusions: ≤2months, allergy, medication interaction)

SDC, Figure 2

Central Line Entry Data Collection form **Date**

Line types (circle all that apply) CVL PICC RA UVC ECMO Other

Line entries for LAB draws

(put an X through the next # each time you make a line entry into any CVL for a lab draw only)

1	2	3	4	5	6	7	8	9	10
11	12	13	14	15	16	17	18	19	20

All other line entries

(put an X through the next # each time you enter any CVL for any reason other than a lab draw ie med, blood return check, syringe change, line change) **Sequences are 1 entry----flush,med,flush,med,flush =1**

1	2	3	4	5	6	7	8	9	10
11	12	13	14	15	16	17	18	19	20

SDC, Figure 3

Disinfection of Needleless Connector Performance Validation

Name/Title________________________________ Unit__________ Date__________

Circle Attempt: #1 #2 #3

The evaluator must be a "Qualified Observer" for this skill and the skill must be within their scope of practice. The evaluation is to be done without coaching to determine if the employee can perform the skill independently. If some critical elements are not met, describe in comments below. Provide feedback, coaching and repeat performance evaluation at a later date.

Please Circle the Methods of Evaluation:

For Competency: Observed in clinical setting during care: **DO**

For Skills Validation: Return Demonstration: **RD;** Written Assessment: **WA;** Simulation: **S;** Reviewed Policy: **RP**

Critical Elements		Met	Not Met
1. Perform hand hygiene.			
2. Don clean gloves.			
3. Place sterile drape under central line, between patient and line. Supplies needed for line access must be on this drape or on approved work surface after disinfection.			
4. Remove alcohol port protector from needleless connector.			
5. Scrub needleless connector with alcohol scrub device for defined time (10 sec=site scrub, 15 sec=alcohol prep pad), and allow to dry for at least 5 sec.			
6. Connect flush/medication.			
**Special Considerations**	Met	Unmet	NA
1. When connecting/disconnecting hold onto the needleless connector near the connection to prevent loosening of needleless connector from central line.			

Remediation Guidelines		
If RN does not meet EVERY critical element listed WITHOUT coaching, the Qualified Observer will review the competency with RN, coach them on how to improve, tell them another Qualified Observer will reevaluate this competency in the future and submit form to the Unit Educator for documentation.	Needed coaching	NA
If a second opportunity for evaluating this competency is needed, the Qualified Observer will complete this form and submit it to the Unit Educator for documentation.	2nd evaluation needed	Manager/ Designee notified
If a THIRD opportunity for evaluating this competency is needed, the Qualified Observer will complete this form, submit it to the Unit Educator for documentation and NOTIFY the Manager or designated individual completing the individual RN's performance appraisal.	3rd Evaluation needed	Manager/ Designee notified

The RN demonstrates the ability to apply knowledge, skill and judgment to safely care for patients in accordance with the above skill.

Qualified Observer Name (Printed): ______________________________

Qualified Observer Name (Signature): ______________________________ Date Completed: ________
